# Specificity Determination in *Saccharomyces cerevisiae* Killer Virus Systems

**DOI:** 10.3390/microorganisms9020236

**Published:** 2021-01-23

**Authors:** Lina Aitmanaitė, Aleksandras Konovalovas, Povilas Medvedevas, Elena Servienė, Saulius Serva

**Affiliations:** 1 Laboratory of Nucleic Acid Biochemistry, Institute of Biosciences, Life Sciences Center, Vilnius University, 10257 Vilnius, Lithuania; lina.aitmanaite@gf.vu.lt (L.A.); aleksandras.konovalovas@gf.vu.lt (A.K.); povilas.medvedevas@gmc.stud.vu.lt (P.M.); 2 Laboratory of Genetics, Nature Research Centre, 08412 Vilnius, Lithuania; elena.serviene@gamtc.lt

**Keywords:** *Saccharomyces* yeast, *Totiviridae*, dsRNA, LA virus, killer virus

## Abstract

*Saccharomyces* yeasts are widely distributed in the environment and microbiota of higher organisms. The killer phenotype of yeast, encoded by double-stranded RNA (dsRNA) virus systems, is a valuable trait for host survival. The mutual relationship between the different yet clearly defined LA and M virus pairs suggests complex fitting context. To define the basis of this compatibility, we established a system devoted to challenging inherent yeast viruses using viral proteins expressed in trans. Virus exclusion by abridged capsid proteins was found to be complete and nonspecific, indicating the presence of generic mechanisms of *Totiviridae* maintenance in yeast cells. Indications of specificity in both the exclusion of LA viruses and the maintenance of M viruses by viral capsid proteins expressed in trans were observed. This precise specificity was further established by demonstrating the importance of the satellite virus in the maintenance of LA virus, suggesting the selfish behavior of M dsRNA.

## 1. Introduction

*Saccharomyces* yeasts are widely distributed in the environment and microbiota of higher organisms, including humans [1,2]. Historically, yeasts were employed in fermentation industries, and current research has made yeast a profitable host for the production of biotechnologically and pharmaceutically relevant proteins [3]. The biocidal property of yeast—known as its killer phenotype—was found to be a highly desirable trait in industrial strains for the restraining of spoilage microorganisms and preservation of the quality of food products and beverages [4]. Yeast’s killer trait is often determined by two viruses, LA, and M, ensuring killer toxin production and maintenance in a host cell. The frequency of killer M viruses among *S. cerevisiae* strains is estimated to be relatively low [5], whereas the LA virus is prevalent in various wild, industrial and laboratory yeasts [6].

The double-stranded RNA (dsRNA) genome of the LA virus encodes the coat protein Gag and RNA-dependent RNA polymerase GagPol. These two proteins assemble into capsids, hosting the replication and transcription of LA itself and satellite M virus to provide a killer phenotype to the host cell [7]. The extracellular phase of LA virus is unknown [8], and the presence of LA alone appears to be symptomless in yeast cells [9]. The amount of 4.6 kbp size LA dsRNA was found comparable to that of cellular rRNA [10], making a significant contribution to the total RNA content. The killer virus M hijacks LA capsids for its maintenance, encoding preprotoxin targeting virus-free cells after the maturation and secretion [7]. This mutual relationship makes LA and M dsRNA viruses functionally linked in precise synergy with the host cell.

Structurally, LA viruses comprise a uniform group featuring a short 5′ untranslated region, followed by a two partially overlapping ORFs and a 3′ untranslated region. The capsid protein Gag is encoded on the 5′ part of the genome, whereas 101 nt from its CDS end, there is a robust secondary structure, a region resulting in a −1 frameshift of the ribosome and synthesis of the fusion protein GagPol [11,12]. M dsRNA features the short 5′ untranslated region followed by ORF of K toxin, synthesized in preprotoxin form, with a 3′ polyA stretch of several hundred nucleotides and a 3′ untranslated region, hosting encapsidation and replication signals [8]. By far the best explored K1 toxin binds to yeast cell envelope glucans, as does the K2 toxin [13,14,15], and both of them form pores in the cytoplasmic membrane of sensitive cells. K28 penetrates into the cell and further into the nucleus to block DNA synthesis [16], whereas the Klus’ mode of action remains to be uncovered.

Several *Totiviridae* LA variants have been described in *Saccharomyces cerevisiae* [17,18,19]. Despite the average 74% nucleotide sequence identity shared by LA viruses, their compatibility with sequence-unrelated M satellites is complex [18,19]. Currently, four LA family viruses, namely LA-1, LA-2, LA-lus and LA-28, have been described to associate with respective M1, M2, Mlus and M28 satellites, although the number of following pairs was found to be larger than four [17,20]. Among the wild-type *S. cerevisiae* strains, LA-1 was found to exclusively maintain the M1 satellite, whereas LA-2 maintains M2 and LA-28 maintains the M28 satellite. LA-lus was reported to maintain either Mlus or M2 in wild-type strains [20]. The association of distinct LA viruses with different M viruses is interpreted to stem from coevolution, driven by the toxin encoded by the satellite virus [17]. *Totiviridae* viruses have moderate impact on host gene expression, yet the transcriptional response to viral dsRNA elimination is broad, supporting the idea of long-lasting coadaption [20,21]. The importance of the host background was revealed by different killer phenotypes of distinct strains bearing dsRNA viruses of the same type, which is also explained by a virus and host coevolution in different populations [5]. Specific *S. cerevisiae* host cell lipidomic and transcriptomic adaptations were found to occur in cells producing the K1 killer toxin [22]. The LA virus appears to be less demanding than the satellite M, since only a few host genes were described as crucial for LA maintenance [8]. In addition, LA tends to be more resilient to elimination than M, suggesting a more vital interconnection with the host [8].

In this study, we took advantage of the current repertoire of *Saccharomyces cerevisiae* killer systems to address the foundations of the specificity within the complete range of LA and M virus pairs. For this, we cloned the representatives of all known *Saccharomyces cerevisiae* LA viruses. A plasmid-based system, devoted to challenging the inherent yeast dsRNA viruses in trans expressing viral proteins from either the same or related viruses, was established. Expression of the abridged capsid protein led to complete and nonspecific virus exclusion within the LA range, pointing to generic mechanisms of *Totiviridae* maintenance in yeast cells. Indications of specificity in both the exclusion of LA viruses and the maintenance of M viruses by viral capsid proteins expressed in trans were observed. The concurrent mode of M1 dsRNA in the maintenance of LA-1 in a wild-type strain was uncovered, suggesting selfish behavior by M dsRNA, with a yet-unknown mechanism.

## 2. Materials and Methods

### 2.1. Strains and Growth Medium

Yeast strains used in this study are summarized in Table 1. Yeast cells were grown in YPD medium (1% yeast extract, 2% peptone, 2% glucose; for plates, 2% of agar was added). For the selection of yeast transformants, YPD medium was supplemented with different G418 concentrations (K7—400 µg/mL; Rom K-100/ SRB-15-4/ M437—200 µg/mL; K28—100 µg/mL). For killer assays, MBA medium was used (0.5% yeast extract, 0.5% peptone, 1.05% citric acid, 3.53% disodium phosphate, 2% glucose, 2% agar, 0.003% methylene blue). The medium was adjusted to pH 4.2. Yeast cells for the purification of viral particles were grown in SD medium (0.67% yeast nitrogen base, 2% glucose). According to need, uracil (10 µg/mL) and amino acids L-leucine (60 µg/mL), L-methionine (10 µg/mL) and L-histidine (10 µg/mL) were added.

### 2.2. Protein Expression Vectors

Expression vectors were constructed following the standard cloning methods. The pYAK-3 plasmid was used as a starting construct [29]. This includes the replication origin of *S. cerevisiae* 2 µm plasmid and the URA3 gene for selection. The KanMX gene from pYM14 (Scientific Research and Development GmbH, Oberursel, Germany) was cloned into pYAK-3, resulting in the pYAK-G vector. The wild-type LA-1 Gag protein expression vector (LA1-Gag-wt) was constructed by cloning the Gag coding sequence (accession no. J04692) to the pYAK-G plasmid under strong constitutive promoter TEF1. Expression vectors of truncated LA-1, LA-2 (accession no. KC677754), LA-lus (accession no. JN819511) and LA-28 Gag (accession no. KU845301) proteins were constructed by cloning partial Gag proteins coding sequences (LA1-Gag-delta and LAlus-Gag-delta – 1-1923 nt; LA2-Gag-delta and LA28-Gag-delta – 1-1932 nt) to the pYAK-G plasmid. The truncated Gag protein’s coding sequences were fused to FLAG-tag, linker GSGGS and HIS-6 coding sequences. To construct the wild-type viral protein expression vectors, the coding sequences of LA-1 (LA1-GagPol), LA-lus (LAlus-GagPol), and LA-28 (LA28-GagPol) viral proteins were cloned to the pYAK-G plasmid. Correct coding sequences of all constructed vectors were confirmed by sequencing.

### 2.3. Yeast Tansformation

Yeasts were transformed using the lithium acetate and polyethylene glycol method [30], with adjustments. The yeast strain was inoculated into 4 mL YPD and grown for 16–20 h at 30 °C on a shaker at 200 rpm. Five hundred microliters of resulting culture were transferred to 50 mL of fresh YPD medium and incubated at 30 °C on a shaker at 200 rpm until it reached OD_600_ 0.7. Yeast cells were collected by 5 min centrifugation at 1000 × g and washed with 2 mL of sterile water. Collected cells were resuspended in 300 µL 1xLiAc/1xTE buffer (100 mM LiAc pH 7.5, 10 mM Tris-HCl pH 7.5, 0.5 mM EDTA). For each transformation, 100 µL of competent yeast cells were mixed with 0.5–1 µg of plasmid DNA and 600 µL of 1xLiAc/1xTE/40% PEG buffer (100 mM LiAc pH 7.5, 10 mM Tris-HCl pH 7.5, 0.5 mM EDTA, 40% PEG 4000). The resulting mixture was incubated for 30 min at 30 °C on a shaker at 200 rpm. Before heat shock, 70 µL of DMSO was added. Heat shock was carried out by heating a sample for 15 min at 42 °C. Cells were collected by brief centrifugation of 10 s at 14,000× *g*, resuspended in 1 mL of YPD medium and incubated for 2 h at 30 °C on a shaker at 200 rpm to allow expression of the resistance gene. After the incubation, cells were collected by brief centrifugation and plated on YPD plates supplemented with G418 for selection.

### 2.4. Total RNA and dsRNA Extraction

Total RNA and dsRNA were extracted as described previously [20].

### 2.5. Purification of Viral Particles

Yeast strain BY4741 transformed by expression vectors of LA-1 abridged (LA1-Gag-delta) and wild-type (LA1-Gag-wt) Gag proteins were grown in SD medium overnight at 30 °C. Cells were harvested by centrifugation at 6000× *g* for 10 min at 4 °C, resuspended in 0.1 part of initial volume of sterile water and pelleted by centrifugation under the same conditions. Yeast were resuspended in lysis buffer (50 mM Tris-HCl pH 7.4, 200 mM NaCl, 1 mM PMSF), 5 mL buffer for 1 g of yeast and ground with glass beads, then centrifugated at 12,000× *g* for 10 min at 4 °C. The collected supernatant was loaded onto a chilled 45% sucrose cushion in centrifuge tubes and ultracentrifuged for 16 h at 70,000× *g* at 4 °C (Kontron TST 28.38 rotor and Sanyo MSE MS60 ultracentrifuge). The supernatant was discarded, and the pellet resuspended in 500 μL lysis buffer without PMSF for further analysis.

### 2.6. TEM Analysis

Electron microscopy was performed using a Morgagni 268 (D) transmission electron microscope (FEI, Hillsboro, OR, USA). First, 0.2 μg of protein of interest was poured on a copper grid coated with carbon. After 1 min, the grids were dried with filter paper, washed with water, and dried again using filter paper. A 2% uranyl acetate solution was applied to the grid for 2 min. The grid was dried with filter paper and additionally dried for 5 min in the air.

### 2.7. Elimination of M1 Virus from K7 Strain

M1 virus elimination from the K7 yeast strain was performed by streaking on YPD agar plates and growing at 37 °C for two days. The individual colonies were streaked on new YPD agar plates and grown at 30 °C for two more days. For killer phenotype examination, the resulting colonies were transferred on MBA plates with an overlay of α‘1 K1-sensitive strain and grown at 20 °C for three days. Colonies not exhibiting the killer phenotype (with absent halos) were selected for total RNA and subsequent dsRNA purification. The elimination of the M1 virus was confirmed by analysis of dsRNA by agarose gel electrophoresis and two-step RT-PCR using primers specific for M1 coding sequence (M1-dir – 5′-GAAAAATAAAGAAATGACGAAGCC-3′; M1-rev – 5′- CCCTAGTGGCCTGTGTCAC-3′).

### 2.8. Two-Step RT-PCR on Total RNA from Strains BY4741, K7 and M437

To confirm the LA-1 and LA-lus virus elimination from BY4741, K7 and M437 strains, two-step PCR was performed. First-strand cDNA synthesis was carried out with a SensiFast cDNA Synthesis Kit (Bioline, Memphis, TN, USA), following the manufacturer’s recommendations. To identify specific LA-1 or LA-lus cDNA sequences, PCR was performed with a Phusion High-Fidelity DNA Polymerase (Thermo Scientific, Vilnius, Lithuania). Primers used for PCR: for LA-1 sequence-specific—LA 1.F – 5’-GATGTTCTCACTCACAAG-3′, LA-1.R – 5′-GCGTCCATTATTCTTACTG-3’; for LA-lus sequence-specific—LA-lus.F – 5′-GATGCATAGAATCAATG-3′, LA-lus.R – 5′-GTAGTGCTCTTAAAGG-3′.

### 2.9. Densitometric Analysis

Densitometric analysis was performed to quantify the relative amount of LA and M virus dsRNA genomes. The gels of total RNA or dsRNA electrophoresis were analyzed using ImageJ software, version 1.52a (National Institutes of Health, Bethesda, MD, USA). The dsRNA amount of LA and M virus for each sample was normalized by 18S rRNA and compared to the values of control samples. For each yeast transformation, three biological replicates with three technical replicates per sample (nine in total) were used.

### 2.10. Statistical Analysis

Statistical analysis was accomplished by R software, version R-3.6.2 (University of Auckland, Auckland, New Zealand). The values obtained from the densitometric analysis were checked for normality using the Shapiro–Wilk test. To examine if values between the control sample and specific transformants differed significantly, we applied Student’s *t*-test. *p*-values below 0.05 were considered statistically significant (* *p* < 0.05, ** *p* < 0.01, *** *p* < 0.001). Box plots were drawn using R package ggplot2.

## 3. Results

To address the compatibility of LA and M viruses, we cloned cDNAs of complete LA dsRNA genomes present in different killer strains and LA-1 dsRNA, present in laboratory strain BY4741 (Table 1). LA dsRNAs were first isolated by phenol treatment, gel-purified and used as a substrate for primer ligation, subsequent reverse transcription and cDNA amplification [19]. LA-1 capsid protein Gag from the K7 strain was selected to prepare its truncated version by replacing the 39 C-terminal amino acids with a 21-aa-long fragment including FLAG-tag, linker GSGGS and the His6 motif. The resulting LA1-Gag-delta was expressed under the control of a strong constitutive TEF1 promoter (Figure S1A). The virions’ structure was verified using ultracentrifugation-based purification of capsid proteins in BY4741 cells with subsequent TEM analysis, which confirmed the production of capsids of the same size as for the LA-1 virus (Figure S1B).

**Figure 1:**
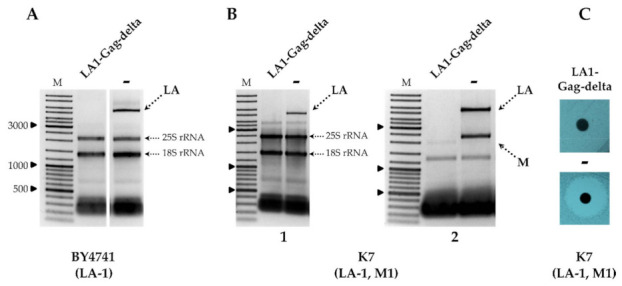
The impact of LA1-Gag-delta overexpression on the maintenance of LA and M dsRNAs. (**A**) Agarose gel electrophoresis of total RNA isolated from the BY4741 strain; LA1-Gag-delta is the sample of the strain transformed by truncated LA‑1 Gag protein expression vector, **−** is the control sample. (**B**) Agarose gel electrophoresis of total RNA (1) and dsRNA (2) isolated from K7 strain transformants. (**C**) Killer phenotype assay of K7 yeast strain transformant. M column is the molecular size marker (GeneRuler DNA Ladder Mix, Thermo Scientific, Vilnius, Lithuania); yeast strains with corresponding genotypes are indicated below the pictures.

Following the transformation of the BY4741 strain by plasmid encoding LA1-Gag-delta, a rapid loss of LA-1 viral dsRNA was observed using agarose gel electrophoresis analysis of total nucleic acids (Figure 1A) and confirmed using RT-PCR (Figure S2). LA-1 dsRNA remained intact in the same strain transformed by the cloning vector lacking LA1-Gag-delta CDS Figure 1A and Figure S2. The loss of LA-1 was definitive— recovery of dsRNA is not observed after the loss of the plasmid coding for LA1-Gag-delta by growing the strain under nonselective conditions in YPD media (not shown).

**Figure 2:**
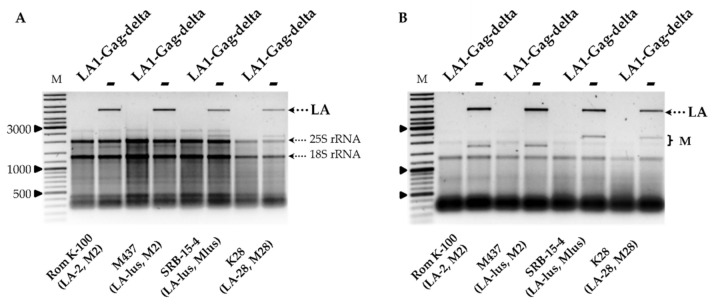
The impact of LA1-Gag-delta overexpression on the maintenance of LA and M dsRNAs in Rom K-100, SRB-15-4, M437 and K28 yeast strains. Agarose gel electrophoresis of total RNA (**A**) and dsRNA (**B**) isolated from respective yeast strain transformants; LA1-Gag-delta refers to samples transformed by truncated LA‑1 Gag protein expression vector, **−** signifies control samples. M column is molecular size marker (GeneRuler DNA Ladder Mix, Thermo Scientific, Vilnius, Lithuania); yeast strains with corresponding genotypes are indicated below the pictures.

Removal of LA-1 dsRNA also occurred in the K1 killer strain K7 (LA-1, M1), as demonstrated in Figure 1B, and was followed by the loss of the killer phenotype, as shown in Figure 1C. In order to address the specificity of the LA-1 exclusion, LA1-Gag-delta was introduced into the wild-type killer strains Rom K-100 (LA-2, M2), SRB-15-4 (LA-lus, Mlus), M437 (LA-lus, M2) and K28 (LA-28, M28). Electrophoretic analysis of total RNA and dsRNA fractions (Figure 2A,B) demonstrated the loss of the corresponding LA dsRNA fraction altogether with M dsRNA, indicating virtually complete clearance of all employed strains from the viral dsRNA genomes. The elimination of LA viruses from BY4741, K7 and M437 strains was verified using RT-PCR (Figure S2) and further confirmed by analysis of killer sensitivity of the resulting strains; expression of LA1-Gag-delta resulted in a loss of killing capacity for all killer strains (not shown).

**Figure 3:**
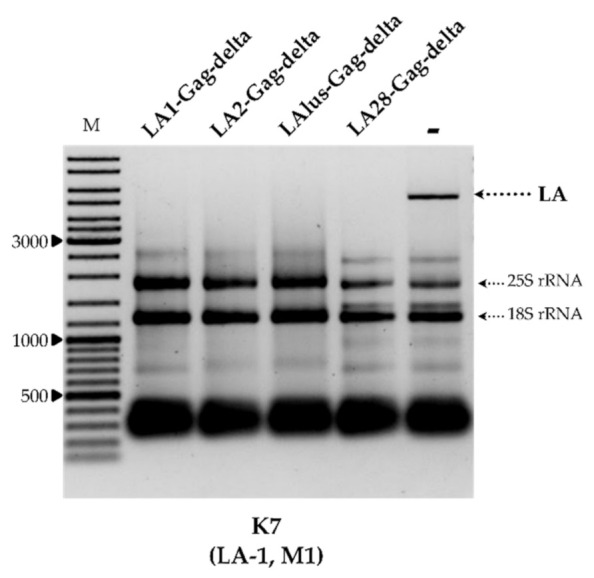
The impact of overexpression of truncated LA-1, LA-2, LA-lus and LA-28 Gag proteins on the maintenance of LA dsRNA in the K7 strain. Agarose gel electrophoresis of total RNA isolated from respective yeast transformants: LA1/LA2/LAlus/LA28-Gag-delta refers to samples transformed by truncated LA Gag protein expression vectors, **−** denotes the control sample. M column is molecular size marker (GeneRuler DNA Ladder Mix, Thermo Scientific, Vilnius, Lithuania); yeast strain with corresponding genotype is indicated below the picture.

To better understand the specificity of dsRNA exclusion, we prepared plasmids encoding the truncated capsid protein Gag originating from LA-2, LA-lus and LA-28 viruses. Given the uniform organization of known *Saccharomyces* spp. LA viruses, the most 5′ CDS was employed. These constructs, and the expression vector used as a control, were introduced into strains BY4741, K7, Rom K-100, M437, SRB-15-4 and K28. The analysis of total RNA and dsRNA content confirmed the complete loss of both LA and M dsRNA fractions, corresponding to loss of the killer phenotype, in all transformed strains. Figure 3 illustrates the total RNA content of the K7 strain transformed by plasmids coding for LA1-Gag-delta, LA2-Gag-delta, LAlus-Gag-delta and LA28-Gag-delta, or an empty expression vector.

**Figure 4:**
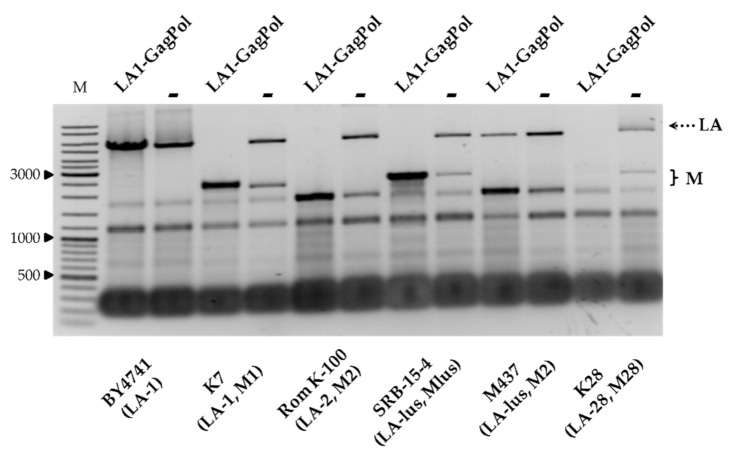
The impact of overexpression of LA-1 capsid proteins on the maintenance of LA and M dsRNAs in BY4741, K7, Rom K-100, SRB-15-4, M437 and K28 strains. Agarose gel electrophoresis of dsRNA isolated from respective yeast strain transformants; LA1-GagPol refers to samples transformed with LA-1 proteins expression vector, **−** denotes control samples. M column is molecular size marker (GeneRuler DNA Ladder Mix, Thermo Scientific, Vilnius, Lithuania); yeast strains with corresponding genotypes are indicated below the pictures.

We next addressed the compatibility of the viral capsid proteins, encoded by the LA-1 virus from the K7 strain, with the inherent *Totiviridae* viruses from the wild-type killer strains. The vector encoding GagPol under the TEF1 promoter was used for transformation of strains BY4741, K7, Rom K-100, SRB-15-4, M437 and K28. Strains considered as controls were transformed by an empty expression vector, grown under the same conditions and processed in parallel. Notably, the amount of LA dsRNA increased in BY4741, lacking M dsRNA (Figure 4). In the killer strains, the amount of LA either diminished (as for M437) or entirely disappeared, as in the K7 and K28 strains. In contrast to LA, the amount of M dsRNA generally increased, with the exception of that from the K28 strain (Figure 4).

**Figure 5:**
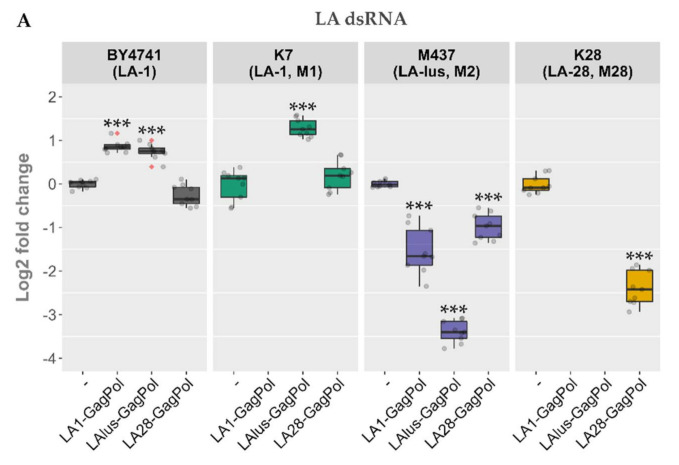

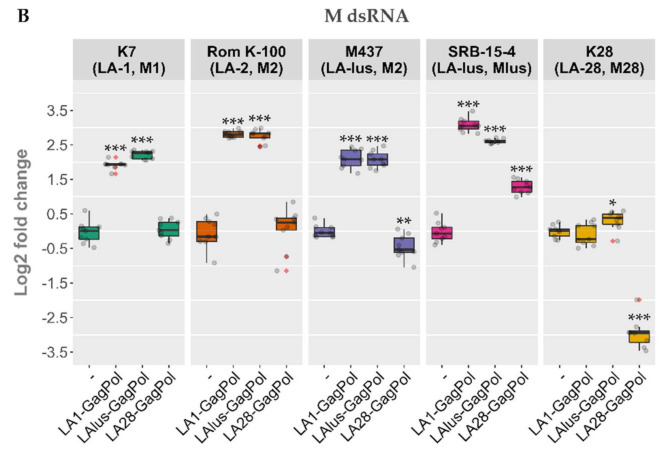
The impact of overexpression of LA-1, LA-lus, LA-28 capsid proteins on the maintenance of LA and M dsRNAs in BY4741, K7, Rom K-100, SRB-15-4, M437 and K28 strains. (**A**) Relative change in the amount of native LA virus dsRNA genome as compared to control samples (-). (**B**) Relative change in the amount of native M virus dsRNA genome as compared to control samples (‑). Examined yeast strains are specified at the top, protein expression vectors used are listed below. Gray dots indicate individual samples, red dots denote outliers. Statistical significance was evaluated using Student’s *t*-test. *p* values below 0.05 were considered statistically significant (* *p* < 0.05, ** *p* < 0.01, *** *p* < 0.001).

Following the same scheme, the expression plasmids bearing LA-lus and LA-28 complete coding sequences were prepared and were used to transform the BY4741 along with killer strains K7, Rom K-100, M437, SRB-15-4 and K28. LA dsRNA was quantified from the total nucleic acid preparation (Figure 5A), whereas the dsRNA fraction was used for M dsRNA quantification for a more precise analysis of dsRNA maintenance (Figure 5B). Three samples from three separate colonies (nine samples for every transformed strain) were used to obtain the nucleic acid preparations and were resolved in agarose gel. Amounts of viral dsRNA were quantified by densitometric analysis and normalized with respect to 18S rRNA. The expression of GagPols from different LA viruses in the BY4741 strain resulted in a different outcome as compared to the killer strains. The amount of LA-1 dsRNA in BY4741 was either increased (for LA1-GagPol and LAlus-GagPol expression) or remained at the same level (LA28-GagPol) (Figure 5A). For killer strains, occasional exclusion of parental LA dsRNA was observed: K7 strain lost LA-1 following the expression of LA1-GagPol, K28 lost LA-28 following the expression of LA1-GagPol or LAlus-GagPol. For other combinations, the trend towards diminishing the amount of the parental LA dsRNA was observed; the only exception was the K7 strain, of which the LA-1 dsRNA level was boosted 2.45-fold by the expression of LAlus-GagPol. Of note, Rom K-100 and SRB-15-4 strains lost inherent LA completely under the expression of any of the employed GagPols—encoded by either LA-1, LA-lus or LA-28—and therefore are omitted from Figure 5A.

Contrary to generally diminished LA dsRNA levels, the expression of capsid proteins led to elevation of the M dsRNA level 3.8–8.5 fold (Figure 5B). LA1-GagPol and LAlus-GagPol displayed the similar effect by boosting M dsRNA levels in K7, Rom, M437 and SRB-15-4 strains; they also had no effect in the K28 strain. In contrast, LA28-GagPol displayed only a moderate increase in the amount of the Mlus dsRNA in the SRB-15-4 strain, while it reduced the amount of the M dsRNA 7.8-fold in the parental K28 strain.

**Figure 6:**
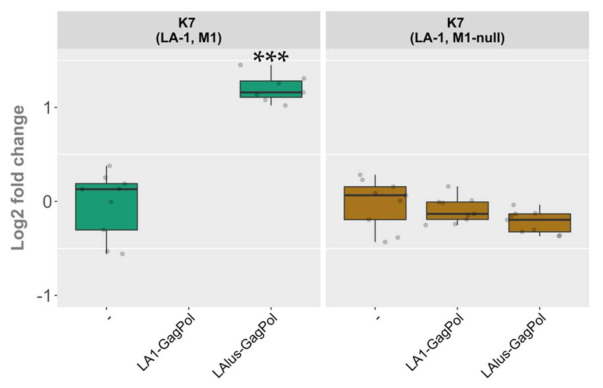
The role of M1 dsRNA in maintenance of the LA-1 virus following the overexpression of LA1-GagPol and LAlus-GagPol proteins. Relative change in the amount of LA dsRNA compared to control samples is depicted (-). Examined yeast strains are specified at the top; protein expression vectors are listed below. Gray dots correspond to individual samples. Statistical significance was evaluated using Student’s *t*-test. *P* values below 0.05 were considered statistically significant (*** *p* < 0.001).

Plasmid-encoded LA1-GagPol and LAlus-GagPol clearly differed in compatibility with the LA-1 present in the K7 strain, though both were able to maintain the M1 dsRNA at comparable rates (Figure 5, panels A and B). Therefore, we decided to address the role of the killer virus present in the K7 strain. We excluded M1 dsRNA from the K7 strain by growing the parental killer strain at 37 °C for several days in a row and selecting killing-negative colonies with LA-1 dsRNA present. The elimination of M1 dsRNA was confirmed using RT-PCR (Figure S3). The M1-cured (M1-null) strain obtained was transformed using LA1-GagPol or LAlus-GagPol expression vectors and compared to the wild-type K7 strain transformed in parallel. Control strains were prepared by transformation with an empty expression vector. In contrast to the wild-type K7 strain, the K7 M1-null strain did not lose the LA-1 dsRNA in the presence of LA1-GagPol expressed in trans (Figure 6 and Figure S3). Moreover, in the K7 M1-null strain the level of LA-1 dsRNA did not increase upon the expression of LAlus-GagPol, in sharp contrast to a 2.45-fold increase seen in the wild-type K7 strain. Strikingly, the expression of this construct in the K7 M1-null strain had virtually the same effect as the expression of LA1-GagPol or the presence of the empty expression vector (Figure 6).

## 4. Discussion

*Saccharomyces cerevisiae* dsRNA LA viruses comprise a group of viruses highly similar by sequence. In biocidic, or killer strains, they are responsible for maintenance of several unrelated satellite M viruses. Killer strains have been found to display a particular connection between LA and M dsRNAs, interpreted as “specificity” between the helper and satellite viruses, respectively. The determinants of such specificity remain unknown and are an object of ongoing debates. Recently, yeast exoribonuclease Xrn1 was proclaimed to undergo positive selection toward totiviruses to enable species-level specificity of antiviral defense mechanisms [31]; however, this finding was questioned by another study [32]. The idea of coevolution of *Totiviridae* LA and M viruses is based on the observation that pair members are functionally linked [18]. Meanwhile, the advances in virus discovery and particularly the use of high throughput sequencing have continued to uncover the range of new entries in the *Totiviridae* family [32,33]. The full landscape of diversity of the LA and M pairs is yet to be discovered. Analysis of individual pair members provides evidence that the former paradigm “one LA–one M satellite” is no longer valid [17,20]. At the same time, previously established and well-grounded coupling of virus pairs indicates the cellular mechanisms capable of ensuring certain viruses’ survival and outcompeting others, if provided in trans [17,18].

It is technically challenging to estimate the functional compatibility of the *Totiviridae* viruses in vivo. We chose to establish a straightforward and manageable plasmid-based system, where inherent viruses would be challenged by in trans expressed viral proteins of any available origin, whether it was the same or a related virus. This approach allowed us to exclude the impact of external cellular factors resulting from cytoplasm fusion, a classic method used to explore the viral compatibility [34,35], and also to take control of the dose of supplied proteins. For the first time, all entries in the current LA virus list identified in *S. cerevisiae* were addressed in order to uncover specificity determinants within the complete range of corresponding LA and M virus pairs. Representatives of genes from all previously known *S. cerevisiae* LA viruses were cloned under the strong constitutive TEF1 promoter, sequenced and expressed. We started by preparing a truncated version of the LA-1 virus capsid protein Gag from the well-described *S. cerevisiae* K7 strain. Previous research reported by [36,37] uncovered the importance of the length of the deletion in the C terminus. Capsid proteins were shown to serve as an elimination factor for the inherent LA-1 virus when truncated by at least 77 amino acids [36]. The circumstances of the virus elimination, however, remained unclear. We observed the exclusion effect of LA-1 Gag, lacking the 39 C-terminal amino acids replaced by a multifunctional 21-aa fragment, and employed it to address the compatibility with other LA family viruses. The expression of this protein in a range of K1, K2, Klus type killer strains and *Saccharomyces paradoxus* K28 resulted in the exclusion of inherent LA and M viruses. Importantly, the same result was observed when similarly engineered Gags originating from LA-2, LA-lus and LA-28 viruses were used. The loss of LA dsRNA was definite, pointing to immediate and irreversible action since no recovery of LA dsRNA was observed after the loss of plasmids and the subsequent cultivation of resulting strain. When GAL1 or weaker ADH1 promoters were used to express the LA1-Gag-delta, various attenuation levels instead of exclusion of LA-1 dsRNA were observed (not shown). TEM investigation of strains expressing Gag-delta (under the control of the TEF1 promoter) confirmed the presence of viral particles resembling those formed by endogenous LA-1. Therefore, dsRNA virus exclusion by the Gag-delta protein appears to be universal and complete under saturating amounts of Gag-delta, presenting a convenient tool to prepare the dsRNA-free *Saccharomyces cerevisiae* and *Saccharomyces paradoxus* strains under the nonmutagenic conditions [20]. The presence of a generic mechanism(s) of *Totiviridae* maintenance, approached by the involved system in a nonspecific fashion, is an obvious interpretation of the observed phenomenon. One apparent possibility for LA exclusion is the ablation of cellular proteins required for viral dsRNA transcription and/or replication by nonfunctional VLP structures formed by a plasmid-borne Gag-delta [36], along with removal of other compounds vital for virus maintenance. Further research on the host factors involved in this process is currently underway.

**Figure 7:**
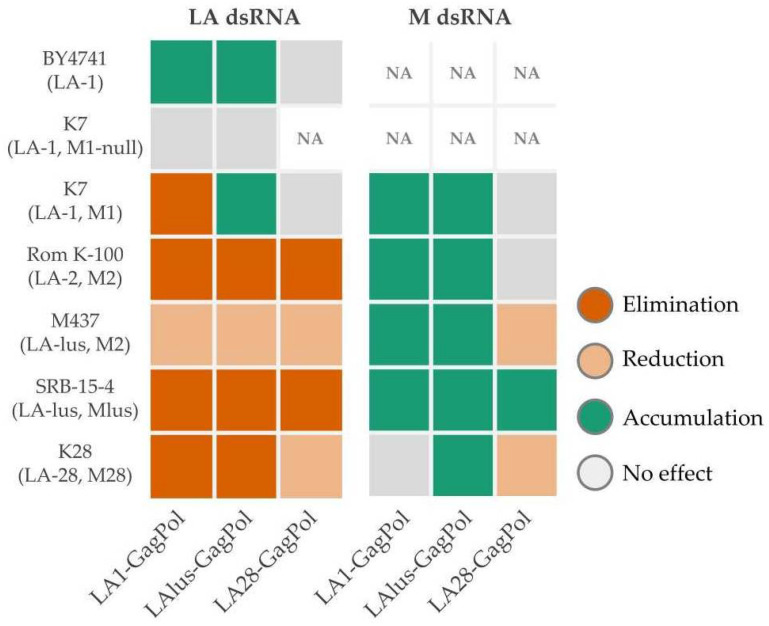
Summary of the LA and M dsRNA amount alterations determined by overexpression of LA-1, LA-lus and LA-28 GagPols. Examined yeast strains specified at the left side of the panel, protein expression vectors listed below.

To investigate the compatibility of inherent dsRNA viruses with heterologous virions, we expressed the full-length GagPol ORFs encoded by LA-1, LA-lus and LA-28 viruses. As established for the LA-1 virus, ribosomal frameshift frequency ensures translation of ~98% of Gag and the remaining ~2% of the fusion GagPol protein [38]. This system allows investigation of the impact of heterologous native virions, providing the transcription and replication-competent environment for both LA and M viruses, addressed in both laboratory and wild-type strains. We observed the general trend for incompatibility of heterologous virions toward inherent LA dsRNA and the opposite—supporting—effect for a M dsRNA (Figure 7). The effects of GagPols from LA-1 and LA-lus on the maintenance of inherent *Totiviridae* viruses were essentially overlapping; notable exceptions are the accumulation of LA-1 dsRNA in the K7 strain, transformed by the LAlus-GagPol expressing plasmid, and the minute change in the amount of M28 dsRNA in the K28 strain, transformed by the LA1-GagPol plasmid. In this context, LA28-GagPol triggers no effect on the presence of LA-1 dsRNA in the K7 strain and the reduction in the LA-28 dsRNA amount in the K28 strain. In strains transformed by the LA28-GagPol plasmid, the accumulation of M dsRNA is found in the SRB-15-4 strain only, with no effect on K7 and Rom K-100 strains and a reduction in the amount observed in M437 and K28 strains. Here, we likely encounter specificity-driven virus cross-fitting. As demonstrated, LA-lus dsRNA present in the M437 strain is generally more resilient to exclusion by any of the GagPols expressed in this strain, including its own LAlus-GagPol. We attribute this effect to the “hybrid” combination of LA-lus and M2 dsRNA in the M437 strain, making virus relations shifted from the general fitting context. The differing sensitivity of LA-1 dsRNA in the K7 strain toward LA-1 and LA-lus GagPols expressed in trans attracted our attention and was explored further, see below for discussion. Although the maintenance of LA-28 dsRNA in the K28 strain generally follows an exclusion trend (except in case of LA28-GagPol overexpression), the amount of M28 dsRNA is either affected insignificantly (LA1 and LAlus GagPol overexpression) or reduced (LA28-GagPol overexpression). We attribute the difference in M28 dsRNA maintenance to species origin—K28 was found to be *Saccharomyces paradoxus*, not the *S. cerevisiae* [20,32]. Therefore, GagPols originating from different viruses appear to display particular specificity in excluding LA viruses and maintaining M viruses. This observation needs to be further verified by the analysis of new *Saccharomyces* killer systems, identified by next-generation sequencing [32,33].

Prompted by the different outcomes of LA-1 and LA-lus GagPols in maintaining the inherent LA-1 dsRNA in the K7 strain, we addressed the role of the M1 killer virus. We prepared an M1-null strain using an established technique involving the cultivation of killer strain K7 at an elevated temperature (+37 °C) and observed the distinct role for M1 dsRNA. In the K7 M1-null strain, the expression of LA-1 and LA-lus GagPols results in essentially the same level of LA-1 dsRNA as in the control strain. In the presence of M1 dsRNA, LA-1 GagPol expels the original virus, while LA-lus GagPol boosts its synthesis 2.45-fold; the M1 dsRNA amount is boosted by 3.8 and 4.65-fold, respectively. The different maintaining capability of the K1 killer phenotype by LA-lus compared to LA-1 was demonstrated previously by [17]; the former is lost if both are combined in one cell. This out-competition was attributed to the relevance of native cellular content [17]; other mechanisms might be inferred as well. In particular, the expression of the plasmid-borne LA-1 GagPol is independent of the inherent LA machinery; therefore, M dsRNA maintenance becomes uncoupled from LA virus-provided capsids. Given the high level of heterogeneous GagPol expressed under the strong TEF1 promoter in the context of the high copy number 2-micron-based plasmid, competition for cellular resources between the inherent virus and an additional replication-competent structure might be significant, resulting in the rapid loss of selective pressure-free counterpart LA-1 dsRNA. Lack of LA-1 dsRNA exclusion in the K7 strain expressing the LA-lus GagPol suggests the presence of nonoverlapping host factor(s), critical for the maintenance of inherent LA virus, and precise specificity in factor selection, allowing the coexistence of both the LA-1 virus and LA-lus GagPol-originated capsids in the same cell. Our data contradicts certain observation of LA-1 and LA-lus incompatibility, as published before [17]. However, the involved strains and expression systems, particularly those for external GagPols, are markedly different for direct comparison. Of note, the stable coexistence of LA-1 and L-BC dsRNA in BY4741 strain and others is well documented [23,39].

We have observed the elevated level of the M1 dsRNA in both LA1-GagPol- and LAlus-GagPol- expressing K7 strains. The loss of LA-1 dsRNA occurs only in the presence of M1 dsRNA; thus, the preference to replicate one substrate over another should be based on inherent features of sequence or structure. In particular, all killer viruses identified so-far possess a polyA stretch, not found in the LA viruses, and constitute a possible selectivity factor, obviously present in plasmid-originated Gag and GagPol mRNAs. Of note is also an elevated level of both LA-1 and M1 dsRNAs in strain with an external LA-lus GagPol. Here we likely encounter a complicated interplay of specificity and availability aspects of host factors, involved in the dsRNA replication machinery. The ability of M1 dsRNA to restrict the copy number of the LA-1 virus has been documented [10]. Even if a lowered amount of LA dsRNA constitutes a price that the host cell pays to acquire the killer phenotype, it is not inevitable—as shown in the case of LA-lus GagPol overexpression in the K7 strain. The alleviation of the LA dsRNA amount determined by the presence of M dsRNA in a cell might result in the clearance of LA, once M gets over-replicated—the exact situation observed for most killer strains featuring heterogeneous virions. Further research is needed to define the relevant cellular factors and to understand the interactions between the helper LA and the satellite M viruses in the native environment. We suggest the presence of mechanisms, ensuring a balance between particular dsRNA replicating structures in the context of availability and specificity of host factors involved in the maintenance of totiviruses in yeast cells.

In summary, we established a plasmid-based system to challenge the inherent yeast dsRNA viruses using viral proteins provided in trans. The exclusion of the viruses by a truncated capsid protein was found to be complete and nonspecific within the tested LA range, pointing to the generic mechanisms of *Totiviridae* maintenance in yeasts. Indications of specificity in both the exclusion of LA viruses and the maintenance of M viruses by heterologous virions were observed. The concurrent role of M1 dsRNA in the maintenance of LA-1 suggests selfish behavior with a yet-unknown mechanism, possessing a precise specificity of action.

## Figures and Tables

**Table 1:** Yeast strains used in this study.

Strain	Species	Genotype	Reference
BY4741	*S. cerevisiae*	MATa, *his3Δ1, leu2Δ0, met15Δ0, ura3Δ0, ScV‑LA‑1*	[23]
K7	*S. cerevisiae*	MATa, *arg9,* [kil-K1], ScV-LA-1, M-1	[24]
K7 M1_null	*S. cerevisiae*	MATa, *arg9,* [kil-K0], ScV-LA-1, M1–0	This study
Rom K-100	*S. cerevisiae*	Wt, HM/HM, [kil-K2], ScV-LA-2, M-2	[25]
M437	*S. cerevisiae*	Wt, HM/HM, [kil-K2], ScV-LA-lus, M-2	[26]
SRB-15-4	*S. cerevisiae*	Wt, [kil-Mlus], ScV-LA-lus, M-lus	[19]
K28	*S. paradoxus*	Wt, [kil-K28], SpV-LA-28, M-28	[27]
a’1	*S. cerevisiae*	MATα, *leu2-2*, [Kil-0]	[28]

## Supplementary Materials

The following are available online at https://www.mdpi.com/2076-2607/9/2/236/s1, Figure S1: Virions extracted from BY4741 (LA-1) strain, transformed by LA-1 wild-type (LA1-Gag-wt) and truncated (LA1-Gag-delta) Gag protein expression vectors in comparison to endogenous LA-1 virions. Figure S2: RT-PCR analysis of total RNA isolated from BY4741, K7 and M437 yeast strains. Figure S3: RT-PCR analysis of total RNA isolated from K7 yeast strains.
