# Supplementary figures and images for: Specificity Determination in Saccharomyces cerevisiae Killer Virus Systems
Source: Microorganisms. 2021 Jan 23;9(2):236. doi: 10.3390/microorganisms9020236 (PMC7912047; doi:10.3390/microorganisms9020236)

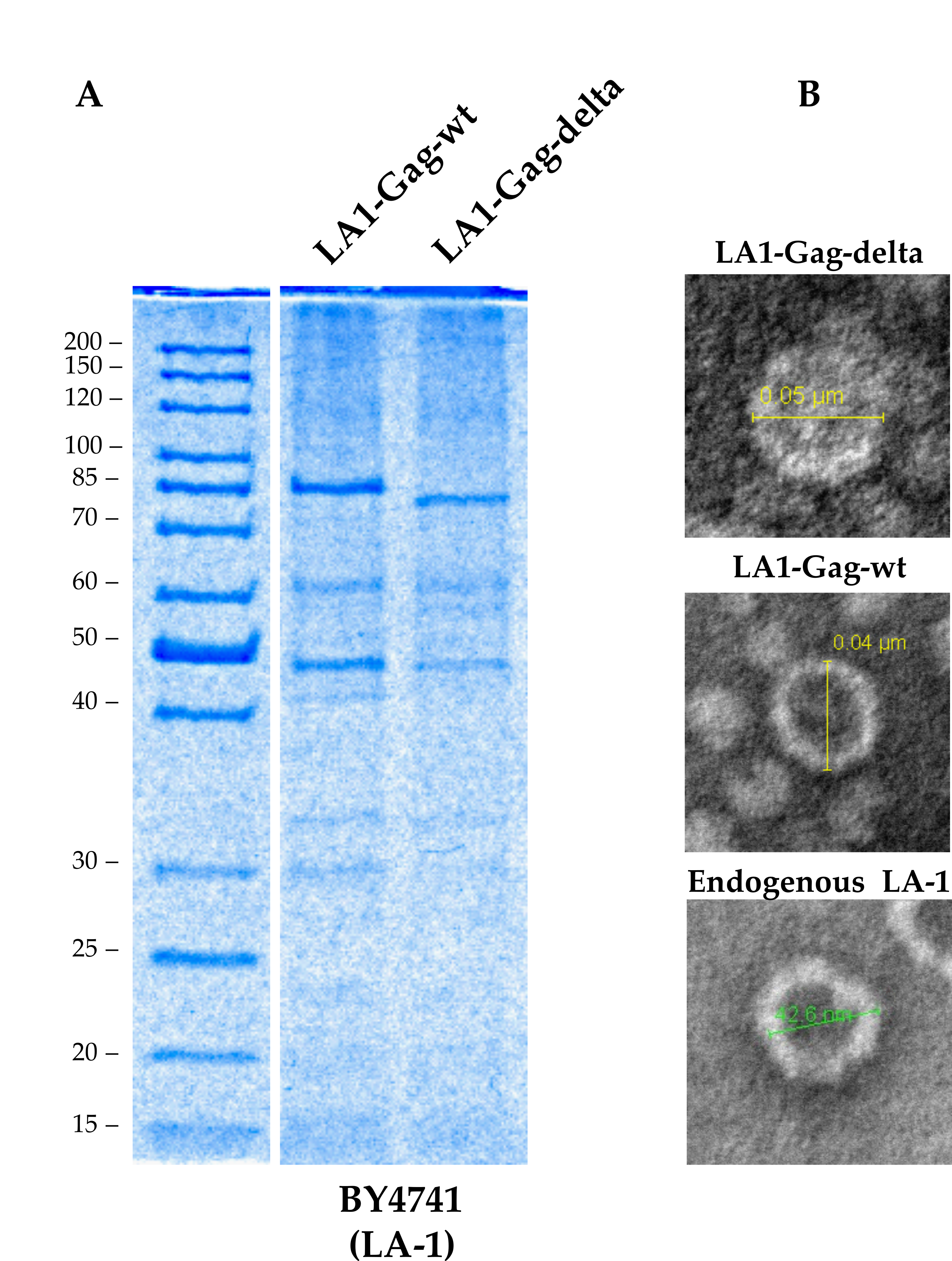

Supplement: Supplementary file 1 [file microorganisms-09-00236-s001.zip › Figure S1.png]

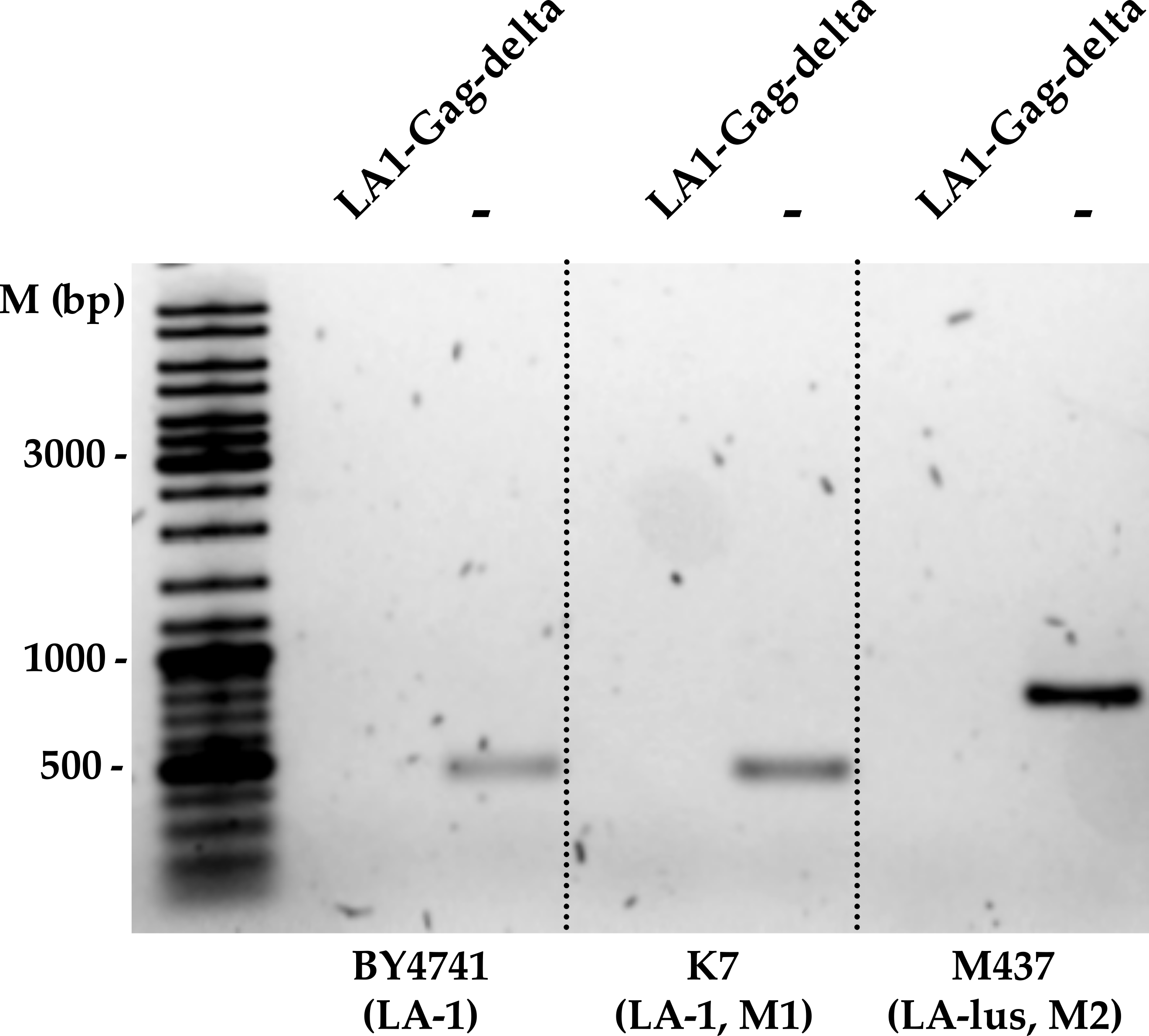

Supplement: Supplementary file 1 [file microorganisms-09-00236-s001.zip › Figure S2.png]

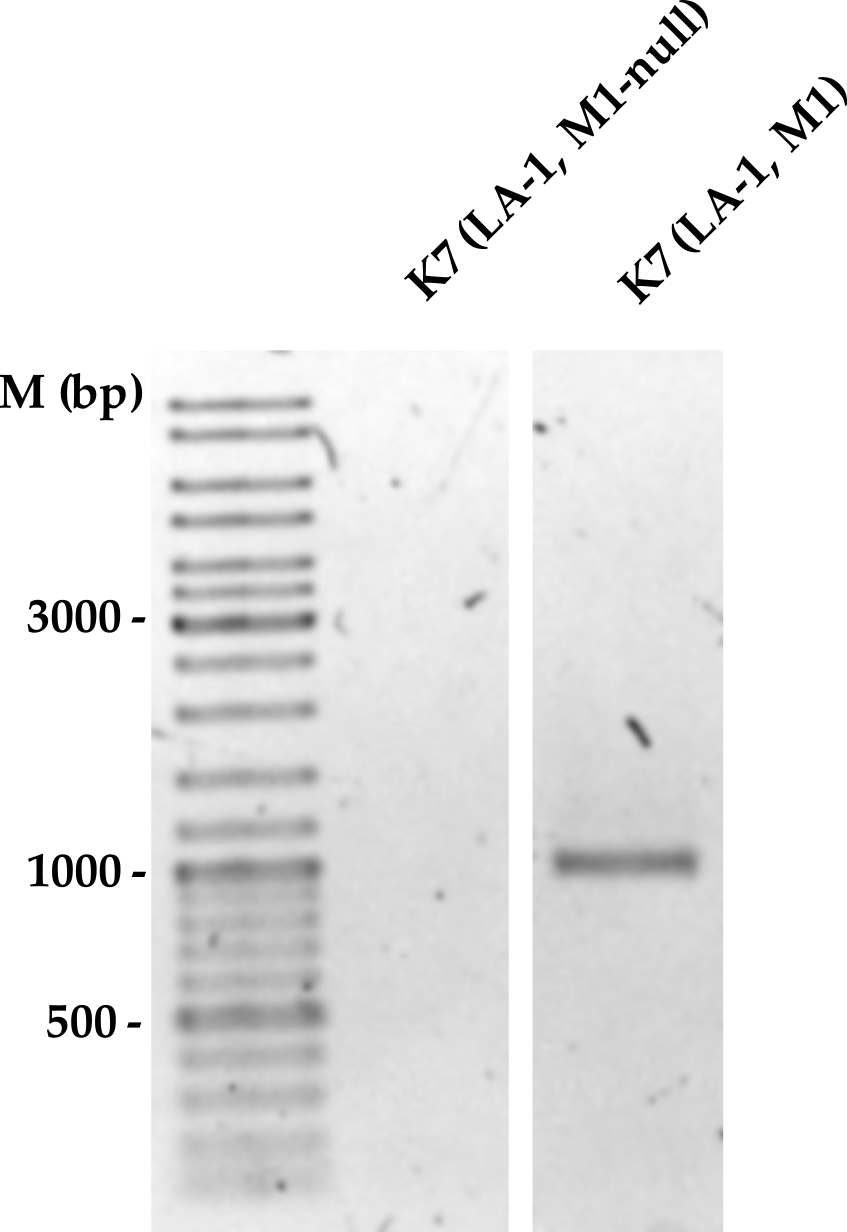

Supplement: Supplementary file 1 [file microorganisms-09-00236-s001.zip › Figure S3.png]
